# 

**Published:** 1915-03-13

**Authors:** 


### CONTENTS.

| | PAGE |
|---|---|
| **The London School of Medicine for Women...** | 523 |
| **Active Service and Medical Officers' Pensions** | 524 |
| **Hospital and Institutional News ... ... ...** | 525 |

A Visit in State to Coventry and Warwickshire Hospital—The German Attack on a Hospital Ship—Black-and-White Signs for Hospitals—The New Children's Hospital, Birmingham—Chairman of the West Kent Hospital Resigns—A Graduated Pay Hospital—Kitchener's Indian Hospital—Mineral - Water Treatment for Officers—The Late Dr. S. H. Habershon—A Testator's Provision for a Nurse—Central Stores for Poor-Law Authorities—Bayoneting an Army Surgeon—Child Welfare Work at Bradford—Birmingham Poor-Law Institutions for the Wounded—Extension of Croydon Mental Hospital—Ex-Patients as Hospital Subscribers—Royal Albert Edward Infirmary, Wigan—Emergency Annual Subscriptions—Aiding the Hospitals' Work for the Wounded—£25,000 for a Cottage Hospital—Contributions for Soldier Patients—"The Education of the Hospital Officer"—Death of Mr. Alexander Hayes—This Week's Drug Market—To Our Readers.

| | PAGE |
|---|---|
| **With the Burgher Commandoes in South Africa ... ... ... ... ... ...** | 531 |

My Medical Experiences in the Campaign: By A Commando Doctor.

| | |
|---|---|
| **Liverpool Royal Infirmary and the Wounded** | 532 |
| **Traumatic Neurasthenia and Mental Disease** | 533 |

The Need for more Precise Definition.

| | |
|---|---|
| **Research and Remedies ... ... ... ...** | 534 |

Urinary Antiseptics.

| | |
|---|---|
| **The Education of the Hospital Officer... ...** | 535 |

Examinations and a Three Years' Course.

| | |
|---|---|
| **Hospital Architecture and Construction ...** | 537 |

Perth Royal Infirmary.

*[See over.]*

### We can supply immediately from stock for Emergency Hospitals

**Bedsteads**
**Mattresses**
**Blankets**
**Linen & Cotton Sheetings**
**Waterproof Sheetings**
**Towellings**
**Flannels**
**Calicoes, &c., &c., &c.**

### D. STEWART & CO.

16 Staining Lane, Gresham Street, London, E.C.

**CONTRACTORS TO HOSPITALS, SANATORIA, &c.**

Telephone: BANK 521. Telegraphic Address: "CENTRALIZE, CENT, LONDON."

### CONTENTS—(*continued*).

| | Page |
|---|---|
| **Buildings Contemplated** ... ... ... ... | 538 |
| **Round the World** ... ... ... ... ... | 539 |
| Fellow-Workers and the Roll of Honour. | |
| **The Evolution of the Modern Hospital** ... | 540 |
| III.—The New Epoch in Equipment and Expense: By B. Burford Rawlings. | |
| **The Department of Modern Nursing** ... ... | 542 |
| IX.—Guy's Hospital: The Preliminary Training School. | |
| **The Editor's Letter-Box** ... ... ... ... | 544 |
| St. Columba's Hospital, Home of Peace. | |
| **Personalia** ... ... ... ... ... ... | 544 |
| **Obituary** ... ... ... ... ... ... ... | 544 |
| **Institutional Needs** ... ... ... ... ... | 544 |
| Stewart's Bedding. | |
| **The Institutional Worker** ... ... (Suppl.) | 1 |
| Institutional Workers and their Work: I. The Hospital Secretary. | |
| Question for March. | |
| Questions Invited. | |
| Answer to February Question: Employers and Workmen's Contributions. | |

